# A Novel Two-Lipid Signature Is a Strong and Independent Prognostic Factor in Ovarian Cancer

**DOI:** 10.3390/cancers13081764

**Published:** 2021-04-07

**Authors:** Liina Salminen, Elena Ioana Braicu, Mitja Lääperi, Antti Jylhä, Sinikka Oksa, Sakari Hietanen, Jalid Sehouli, Hagen Kulbe, Andreas du Bois, Sven Mahner, Philipp Harter, Olli Carpén, Kaisa Huhtinen, Johanna Hynninen, Mika Hilvo

**Affiliations:** 1 Department of Obstetrics and Gynecology, Turku University Hospital and University of Turku, 20521 Turku, Finland; litusa@utu.fi (L.S.); sakari.hietanen@utu.fi (S.H.); mijohy@utu.fi (J.H.); 2 Department of Gynecology, Charité—Universitätsmedizin Berlin, Corporate Member of Freie Universität Berlin, Humboldt Universität zu Berlin, and Berlin Institute of Health, Campus Virchow Klinikum, 13353 Berlin, Germany; elena.braicu@charite.de (E.I.B.); jalid.sehouli@charite.de (J.S.); hagen.kulbe@charite.de (H.K.); 3 Zora Biosciences Oy, 02150 Espoo, Finland; mitja@laaperi.com (M.L.); antti.jylha@thl.fi (A.J.); 4 Satasairaala Central Hospital, Department of Obstetrics and Gynecology, 28500 Pori, Finland; sinikka.oksa@satasairaala.fi; 5 Department of Gynecology and Gynecologic Oncology, Kliniken Essen-Mitte, 45136 Essen, Germany; prof.dubois@googlemail.com (A.d.B.); p.harter@gmx.de (P.H.); 6 Department of Obstetrics and Gynecology, University Hospital, LMU Munich, 80337 Munich, Germany; sven.mahner@med.uni-muenchen.de; 7 Institute of Biomedicine and FICAN West Cancer Centre Cancer Research Unit, University of Turku, 20521 Turku, Finland; olli.carpen@helsinki.fi (O.C.); kaisa.huhtinen@utu.fi (K.H.); 8 Department of Pathology and Research Program in Systems Oncology, University of Helsinki and Helsinki University Hospital, 00014 Helsinki, Finland

**Keywords:** ovarian cancer, lipidomics, lipid, prognosis, ceramide, phospholipid, plasmalogen, biomarker, patient stratification, outcome, personalized medicine

## Abstract

**Simple Summary:**

Most ovarian cancer patients initially show a response to primary treatments, but the development of refractory disease is a major problem. Currently, there are no blood-based prognostic biomarkers, and the prognosis of a patient is determined by the International Federation of Gynecology and Obstetrics (FIGO) stage and residual disease after cytoreductive surgery. In this study, we developed and validated a novel test based on the ratio of two circulatory lipids that enables the prognostic stratification of ovarian cancer patients at the time of diagnosis, prior to any oncological treatments. The translational relevance of this test is to find those patients with poor prognosis early on, and to identify patients that are at high risk of recurrence despite complete cytoreduction. Thus, the test enables the early direction of novel targeted therapies to those ovarian cancer patients at greatest risk of recurrence and death.

**Abstract:**

Epithelial ovarian cancer (EOC) generally responds well to oncological treatments, but the eventual development of a refractory disease is a major clinical problem. Presently, there are no prognostic blood-based biomarkers for the stratification of EOC patients at the time of diagnosis. We set out to assess and validate the prognostic utility of a novel two-lipid signature, as the lipidome is known to be markedly aberrant in EOC patients. The study consisted of 499 women with histologically confirmed EOC that were prospectively recruited at the university hospitals in Turku (Finland) and Charité (Berlin, Germany). Lipidomic screening by tandem liquid chromatography–mass spectrometry (LC-MS/MS) was performed for all baseline serum samples of these patients, and additionally for 20 patients of the Turku cohort at various timepoints. A two-lipid signature, based on the ratio of the ceramide Cer(d18:1/18:0) and phosphatidylcholine PC(O-38:4), showed consistent prognostic performance in all investigated study cohorts. In the Turku cohort, the unadjusted hazard ratios (HRs) per standard deviation (SD) (95% confidence interval) were 1.79 (1.40, 2.29) for overall and 1.40 (1.14, 1.71) for progression-free survival. In a Charité cohort incorporating only stage III completely resected patients, the corresponding HRs were 1.59 (1.08, 2.35) and 1.53 (1.02, 2.30). In linear-mixed models predicting progression of the disease, the two-lipid signature showed higher performance (beta per SD increase 1.99 (1.38, 2.97)) than cancer antigen 125 (CA-125, 1.78 (1.13, 2.87)). The two-lipid signature was able to identify EOC patients with an especially poor prognosis at the time of diagnosis, and also showed promise for the detection of disease relapse.

## 1. Introduction

Lipids play a fundamental role in the function of normal cells. They enable chemical energy storage, cellular signaling, cell–cell interactions in tissues, and adequate function of cell membranes, subsequently regulating cell survival, proliferation, and death [1]. These mechanisms are also tightly associated with and modified in oncogenic processes, particularly cell transformation, tumor progression, and metastasis [1,2]. As an emerging hallmark of cancer [3], metabolic and lipidomic dysregulation has attracted scientific interest, and there is increasing evidence on the utility of lipidomics in the discovery of circulating cancer biomarkers as well as disease mechanism exploration in tumors [4].

Comprehensive circulatory metabolic and lipidomic alterations have been described in epithelial ovarian cancer (EOC) [5], which is a malignancy with a generally unfavorable outcome. With a five-year survival expectancy below 50%, EOC still has the highest mortality among gynecological cancers, despite the recent advances in oncological treatments [6]. Although most patients initially show response to primary treatment, the frequent development of refractory disease is a major problem. Cancer antigen 125 (CA-125) is a well-validated biomarker used in the diagnostics and treatment monitoring of EOC; however, it has remained of little utility in the prognostic evaluation of EOC patients in the clinical setting [7]. As circulatory prognostic biomarkers are lacking, the prognostic stratification of patients is currently based on the International Federation of Gynecology and Obstetrics (FIGO) stage and residual disease after cytoreductive surgery [8]. In addition, patients with mutations in the homologous recombination repair (HR) genes have been shown to possess a better prognosis than those without mutations [9]. As of now, targeted therapies, i.e., poly (ADP-ribose) polymerase (PARP) inhibitors and anti-vascular endothelial growth factor (VEGF) monoclonal antibodies, have become part of the standard treatment regimen of EOC patients and consequently, patient selection and the timely administration of targeted treatments have emerged as new challenges in clinical care [10,11]. 

The investigation of circulatory lipidomic changes/aberrations may enable the prognostic evaluation of cancer patients. Recently, a distinct plasma three-lipid signature (ceramide, sphingomyelin, and phosphatidylcholine) was associated with the poor overall survival of patients with castration-resistant prostatic cancer [12]. Equivalently, two ceramide species and 14 phospholipids quantified from the plasma of patients with liver cancer were associated with increased mortality in another contemporary study [13]. Studies evaluating the feasibility of lipidomics in the prognostic stratification of EOC patients are scarce but promising. Specifically, unsaturated phospholipids and ceramide species have been suggested to play important and complex roles in EOC patient outcomes [14,15]. Phospholipids have been shown to augment ovarian cancer invasion and metastasis, i.e., by activating proteolytic enzymes, while ceramides are known to form more complex sphingolipids, which are bioactive lipids suspected to boost the survival of cancerous cells and facilitate tumor progression [16,17]. In the current study, we set out to evaluate the prognostic utility of a novel two-lipid signature test in patients with EOC. The test builds on the ratio of a circulatory ceramide (d18:1/18:0) (Cer(d18:1/18:0)) and a plasmalogen (PC(O-38:4)), of which the former has previously been detected in increasing and the latter in decreasing concentrations in the sera of EOC patients [15]. In addition, the ability of the two-lipid signature test to detect early disease recurrence was evaluated with longitudinal lipid measurements. 

## 2. Materials and Methods

### 2.1. Patient Cohorts and Samples

Global lipidomic analysis was performed for serum samples obtained in 3 independent ovarian cancer study cohorts, 1 from the Turku University Hospital (Turku, Finland) and 2 from the Charité University Hospital (Berlin, Germany) (Table 1). In the Turku cohort, patients with histologically confirmed invasive EOC were prospectively recruited at the University Hospital of Turku, Turku, Finland, in 2009–2019 (ClinicalTrials.gov identifier: NCT01276574). In addition, 114 patients with histologically confirmed benign gynecological diseases (benign tumors, inflammatory processes, and endometriosis) were included in the study. A team of gynecologic oncologists evaluated patients diagnosed with ovarian cancer, and if the tumor was considered primarily unresectable, patients received neoadjuvant chemotherapy with subsequent interval debulking surgery [18]. Finally, a set of 20 high-grade serous carcinoma (HGSOC) patients were selected from the Turku cohort for the longitudinal lipidomic analyses (Table S1). For these patients, samples were collected before each cycle of chemotherapy and during follow up until first relapse.

The cohorts from Charité were prospectively included in the Tumor Bank Ovarian Cancer (www.toc-network.de, accessed on 6 April 2021). The patients in the first Charité cohort (Charité 1) also participated the LION (Lymphadenectomy in Ovarian Neoplasms) clinical trial [19], and consisted exclusively of stage III, completely cytoreduced patients (Table 1). The second cohort from Charité (Charité 2) (Table 1) was a patient cohort where the prognostic serum samples were obtained either before or after neoadjuvant chemotherapy, whereas all the samples for the prognostic analyses from Turku and Charité 1 cohorts were obtained before any oncological treatments. For additional validation, we used baseline serum lipidomics data from a third, previously published [15,20] Charité cohort, Charité 3 (Table 1). 

For all cohorts, the FIGO stage was determined according to the FIGO 2014 guidelines. The operating team carefully assessed the amount of residual disease after cytoreductive surgery, if present. The primary chemotherapy regimen consisted of carboplatin and taxane. The response to primary treatment and progression were defined according to the Response Evaluation Criteria in Solid Tumors (RECIST) guidelines [21]. Second-line medical treatment was chosen individually for each patient based on the timing of the relapse (platinum sensitive vs. resistant) and included chemotherapy indicated for the treatment of relapsed EOC [11]. The progression-free survival (PFS) was calculated from the time of diagnosis to disease relapse.

### 2.2. Lipidomic and Conventional Biomarker Analyses

A global lipidomic screening method was used to analyze the samples. In brief, 10 µL of samples were used for the extraction of the lipids using a modified Folch extraction [22]. The analysis was performed on a hybrid triple quadrupole/linear ion trap mass spectrometer (QTRAP 5500, AB Sciex, Concords, Canada) equipped with ultra-high-performance liquid chromatography (UHPLC) (Nexera-X2, Shimadzu, Kyoto, Japan). Chromatographic separation was performed on an Acquity BEH C18, 2.1 × 50 mm id. 1.7 µm column (Waters Corporation, Milford, MA, USA). The data were collected using a scheduled multiple reaction monitoring (sMRM™) algorithm [23]. The lipidomic data were processed using Analyst and MultiQuant 3.0 software (AB Sciex), and the area or height ratios of the analytes and their corresponding internal standard peaks were normalized with the IS amount and the sample volume. The details of the chromatography and mass spectrometry conditions have been described previously [15]. 

For patients recruited at the Turku University Hospital, the serum CA-125 (U/mL) concentrations were determined from serum samples with the electrochemiluminescence method on the Cobas e 601 instrument or a Modular E170 automatic analyzer (Roche Diagnostics GmbH, Mannheim, Germany). For Charité patients, CA-125 was measured using Elecsys CA 125 II assay (Roche Diagnostics GmbH, Mannheim, Germany). The serum human epididymis protein 4 (HE4) (pmol/L) concentrations were determined with the enzyme immunoassay method according to the instructions of the manufacturer (Fujirebio Diagnostics Inc., Malvern, PA, USA).

### 2.3. Statistical Analyses

Baseline characteristics of the cohorts were described using medians (interquartile range, IQR) for continuous variables. Two-group comparisons were performed by calculating the mean relative difference between the groups, and the *p*-values were determined by parametric *t*-tests on log-transformed concentrations. The selection procedure for the prognostic lipid ratio has been described in detail in Figure S1. Uni- and multivariate Cox proportional hazard regression models were used to determine hazard ratios (HRs) and 95% confidence intervals for the associations of lipids and clinical measurements with overall and progression-free survival of the patients. The effects were expressed per increase in standard deviation of the biomarkers. The changes over time were investigated using linear mixed models with random intercepts, using the lme4 (version 1.1–23) package. R version 4.0.2 was used for all statistical analyses. 

## 3. Results

### 3.1. Selection of a Prognostic Two-Lipid Signature

We carried out a global lipidomic analysis of pretreatment serum samples from 197 ovarian cancer patients and 114 benign controls (Turku study, Table 1). The results were in line with previous findings showing that ovarian cancer profoundly affects the lipidome, and that the lipid alterations are already observable in early-stage patients (Table S2). Furthermore, the results ratified that a large number of the lipids is associated with the overall and progression-free survival of the patients (Table S2), and from these a single prognostic lipid ratio was constructed. The selection was performed by taking from lipids showing association with ovarian cancer and survival, a lipid ratio that showed highest C-statistic for overall survival (Figure S1, Table S2). The lipids selected for this two-lipid signature were PC(O-38:4) and Cer(d18:1/18:0), and the Cer(d18:1/18:0)/PC(O-38:4) ratio showed higher C-statistics for both overall and progression-free survival than these lipids individually (Table S2).

### 3.2. Prognostic Value of the Two-Lipid Signature Test

Cox proportional hazards models for overall and progression-free survival were constructed in all study cohorts to evaluate the prognostic performance of the Cer(d18:1/18:0) / PC(O-38:4) lipid signature when measured prior to surgery or any other oncological treatments. For overall survival, the point estimates for HRs per standard deviation ranged from 1.40 to 2.12 and the C-statistic from 0.592 to 0.707 in the full study cohorts (Turku, Charité 1, Charité 2, Charité 3), as well as in the subcohorts of patients without macroscopic residual disease after surgery (R0) (Table 2). Adjusted HR point estimates varied from 1.10 to 2.16, and the C-statistic from 0.626 to 0.735. Importantly, pretreatment CA-125 value was not indicative of overall survival in any of the study cohorts (Table 2).

For progression, the HR point estimates were between 1.22 and 1.53 in the cohorts, and the C-statistic from 0.524 to 0.644 (Table 2). A C-statistic of 0.615 was recorded for the Charité 1 cohort which included only stage III R0 resected patients (Table 2). Again, CA-125 values were more modest, ranging from 0.418 to 0.592 (Table 2). In the Turku study we had HE4 data available for the majority of the patients, and this clinically established biomarker also showed worse performance than the two-lipid signature, except for the R0 population (Table S3).

### 3.3. Risk Tables for Ovarian Cancer Patients

**Figure 1:**
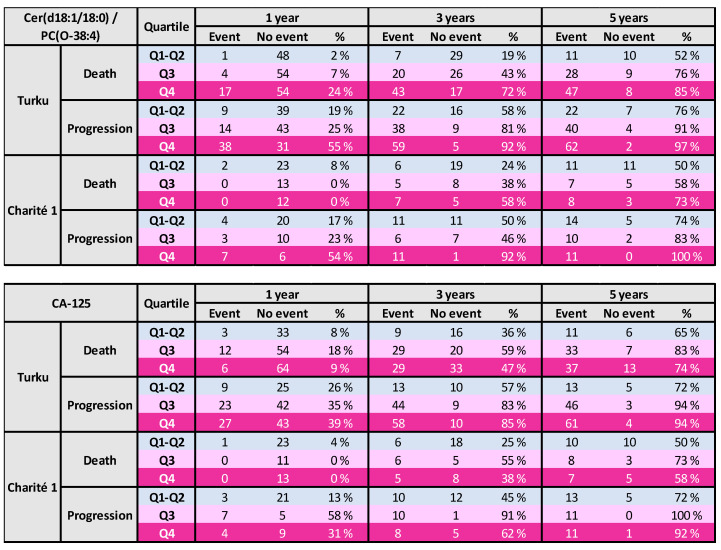
Survival in different quartiles (Q1–Q4) of the lipid ratio (**upper panel**) or CA-125 (**lower panel**).

To illustrate the clinical relevance of the two-lipid signature, patients were split by quartiles of this ratio. The two lowest quartiles were combined to place focus on the highest quartiles. Event rates in these quartiles were calculated at 1, 3, and 5 years in the Turku as well as Charité 1 cohorts. It was apparent that within one year of the diagnosis, the lipid signature already showed consistent risk prediction for progression-free survival, and the difference between the lowest and highest quartiles remained until the five-year follow-up (Figure 1). For death, the separation of the risk in the Charité 1 study became apparent only after three years, whereas it was already evident in the Turku cohort during the first year (Figure 1). For comparison, CA-125 was categorized similarly and in general the results were less consistent than for the lipid ratio (Figure 1).

**Figure 2:**
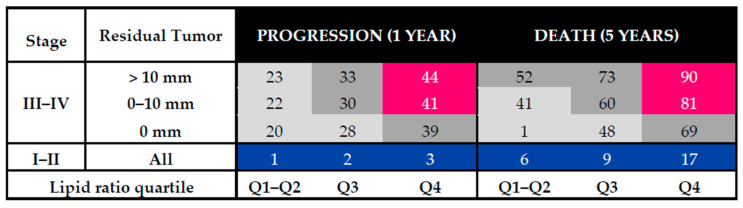
Risk (%) of progression in 1 year or death in 5 years, based on the lipid ratio quartile, stage, and success of tumor removal in surgery in the Turku cohort.

Based on the Turku cohort data, a Cox regression model incorporating lipid ratio quartiles and the clinically important risk factors, i.e., FIGO stage and residual tumor after surgery, was constructed to predict the risk of progression after one year and the risk of death within five years of the diagnosis and biomarker measurements. As expected, for stage I–II tumors the risk was generally much lower than for stage III–IV tumors (Figure 2). However, for this population, after five years the risk of death was almost three-fold higher in the highest quartile of the lipid signature when compared to the lowest quartiles. Importantly, the stage III–IV R0 resected patients belonging to the highest biomarker quartile had a higher risk for both worse progression-free and overall survival than patients with a suboptimal surgery result but belonging to the low biomarker quartiles (Figure 2). When only HGSOC patients were included, the results were similar except for stage I–II patients that had higher overall risk (Figure S2).

### 3.4. Results From Longitudinal Analyses

**Figure 3:**
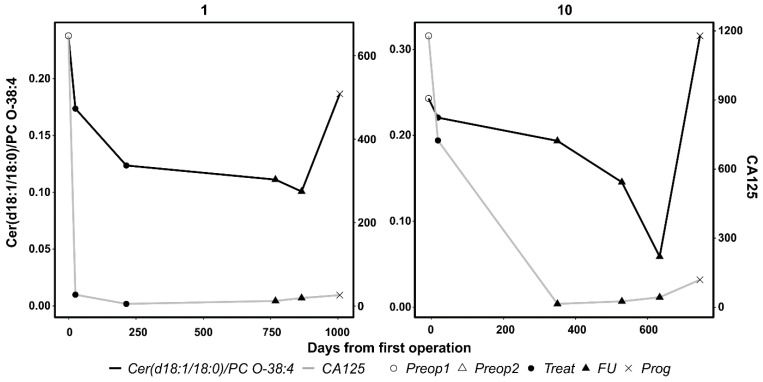
Longitudinal lipidomic and CA-125 analyses of two exemplified ovarian cancer patients. At progression, the two-lipid signature was strongly elevated whereas CA-125 showed none or minor elevation. Preop1, before first surgery; Preop2, before second surgery; Treat, on-treatment; FU, follow-up; Prog, progression.

For 20 patients of the Turku cohort, we analyzed serum samples taken at the time of diagnosis and in different phases during the course of the disease. The results from the linear mixed models analyzing the interaction of biomarkers and possible progression of the disease revealed that the two-lipid signature was more consistently elevated at the time of progression (beta per SD increase 1.99 (95% confidence interval (CI) 1.38, 2.97)) than CA-125 (beta per SD increase 1.78 (95% CI 1.13, 2.87)). Indeed, when the results were plotted for all the patients, it was evident that for two cases (subjects 1 and 10) the lipid signature was clearly elevated at the time of progression, whereas CA-125 showed no or minor elevation (Figure 3 and Figure S3). Importantly, there were no cases where CA-125 had shown elevation during progression but the lipid signature did not (Figure S3).

## 4. Discussion

As of now, prognostic tools for evaluating the outcome of EOC patients are limited to clinical and histopathological variables, while no biomarkers are in routine use. In the current study, we introduced for the first time a two-lipid signature in the prognostic stratification of EOC patients. For cardiovascular risk prediction, it has been shown that distinct lipid ratios outperform single lipid concentrations in the prognostic performance [24,25]. For this reason and to construct as simple a test as possible, we decided to select a single lipid ratio for an ovarian cancer prognostic test. Above all, the lipid test identified especially poor-outcome patients at the time of diagnosis, before any oncological treatments. Unsurprisingly, CA-125 was not significantly associated with the survival of EOC patients.

Patient selection and the most advantageous implementation of precision medicine is of increasing importance in the regular treatment of EOC patients. In ovarian cancer patients with HGSOC histology, a homologous recombination deficiency (HRD) indicates better prognosis and response to platinum and PARP inhibition therapies [26]. Regarding antiangiogenic agents, there is currently no clear consensus on which patients should receive it and more importantly, which patients should not, although bevacizumab is generally seen as a part of the standard treatment of EOC patients [11]. The strength of the current two-lipid signature test is the capacity of finding the poor prognostic patients early: notably, the test functioned in the Charité 1 cohort with completely surgically debulked stage III patients. When identified early at diagnosis, the poor prognosis patients can be offered comprehensive genetic testing and targeted therapies within clinical trials. Altogether, a better prognostic evaluation of individual EOC patients could aid clinicians in directing these treatments more effectively and also improve patient counselling as the modern surgical and oncological treatments are utterly demanding. 

In all patients with EOC, major effort is directed at reaching optimal cytoreduction (0 mm residual disease) as the presence of macroscopic residual disease has been associated with an especially poor prognosis [27]. Indeed, complex ultra-radical surgery including peritonectomy and multiorgan resections have become conventional as the prognostic benefit is generally considered to outweigh the increase in perioperative complications and morbidity [28]. Intriguingly, in the present study, optimally cytoreduced patients in the highest lipid test quartile had an inferior survival outcome compared to patients in the lower lipid test quartiles, regardless of the presence of macroscopic residual disease. These findings suggest that the tumor biology and/or composition of the non-macroscopic residual tumor may have an equally important role in prognosis as the surgery outcome. Of note, in addition to PC(O-38:4), decreased concentrations of a large number of other plasmalogen lipids showed prognostic value. We have previously shown that downregulation of the peroxisome-associated *ABCD1* (ATP Binding Cassette Subfamily D Member 1) gene as well as altered serum lipids and metabolites related to peroxisomal disorders are associated with poor survival in ovarian cancer patients [15,20]. Since the biosynthesis of plasmalogens occurs in peroxisomes [29], it is possible that peroxisomal dysfunction in ovarian cancer cells might explain the downregulation of plasmalogens. In our previous study it was revealed that the alterations in serum ceramides, i.e., their increase or decrease, is dependent on the fatty acyl side chain, and Cer(d18:1/18:0) showed the strongest elevation due to ovarian cancer [15]. Intriguingly, our present data showed that this same ceramide lipid is also the most prognostic one, implying that Cer(d18:1/18:0) is the most important ceramide both diagnostically and prognostically. Thus, it appears that instead of global ceramide upregulation, the alterations are lipid-specific. The possible peroxisomal dysfunction as well as the biological role of Cer(d18:1/18:0) in ovarian cancer warrant mechanistic studies. Further studies on the treatment response are required, as rendering a patient a non-responder for operative treatment a priori is unjustified without robust evidence.

The potential of the two-lipid signature test to detect disease recurrence was estimated in a proof-of-concept manner; however, the exploratory results of this study suggest that aberrations in the concentration of circulatory lipids are already present early-on in tumor development. Indeed, the two-lipid signature test might improve the follow up and early detection of disease recurrence, although it is unclear whether the treatment of early, asymptomatic recurrence is beneficial in the era of novel targeted therapies. It is tempting to hypothesize that the two-lipid signature test might similarly improve the detection of early stage EOC.

The strength of this study is the robust study configuration, as the results were tested in one cohort and validated in three additional, separate cohorts. The LION trial implemented a strict study protocol, which emphasizes the independent prognostic value of the two-lipid signature test. A limitation of the study is that the HR status was not available from our study sets, and the correlation of the lipid test and HRD remains to be assessed in future studies. In addition, the exploration of multiomic profiling might be another feasible method to increase the prognostic potential of the lipidomic analyses [30]. Another limitation is the low number of patients included in the longitudinal analyses; however, the longitudinal analyses were carried out in a proof-of-concept manner and the results need to be further validated in larger patient cohorts. In addition, the lipidomic method utilized is only semi-quantitative, and for clinical use a fully quantitative and analytically validated method has to be developed. However, for one component of the lipid signature, i.e., Cer (d18:1/18:0), a clinically validated method already exists [31], and the addition of another lipid component to the method is feasible. A quantitative method is also needed to confirm the calibration of the risk models between different study cohorts.

## 5. Conclusions

EOC continues to present a challenge for clinicians and scientists alike. Novel, robust, and affordable biomarkers are needed to improve the detection and monitoring of the disease, and also for the optimal allocation of precision drugs and complex surgical treatment. The analysis of circulatory lipids presents a non-invasive method, which differentiates patients with an especially aggressive disease from those with a more favorable disease outcome. Thus, the lipid signature may serve as a novel tool for treatment stratification, which will be an important topic for future studies.

## Figures and Tables

**Table 1:** Clinical characteristics of the study cohorts.

Characteristic	Subgroup	Turku	Charité 1	Charité 2	Charité 3
Malignant (*N*)		197	51	104	147
Age (years)		66 (59–72)	61 (56–68)	65 (57–70)	59 (50–67)
Histology	Serous	156	48	95	147
	Mucinous	13		1	
	Endometroid	16	1	2	
	Clear-cell	12		1	
	Other/unknown		2	5	
Stage	I	31			2
	II	12		1	5
	III	102	51	67	99
	IV	50		24	31
	NA	2		12	10
Follow-up time (years)	Death	2.6 (1.5–3.9)	3.6 (1.9–6.0)	1.6 (0.9–2.3)	3.2 (1.7–4.3)
	Progression	1.3 (0.8–2.2)	2.0 (1.0–3.6)	1.2 (0.8–1.8)	1.5 (0.8–2.8)
Benign (*N*)		114			98
Age (years)		55 (45-68)			41 (31–55)

**Table 2:** Hazard ratios of the two-lipid signature and cancer antigen 125 (CA-125) for overall and progression-free survival.

Endpoint	Study	(Sub)Group	Cer(d18:1/18:0)/PC(O-38:4)						CA-125					
			Ev+	Ev−	HR (95% CI) ^a^	C-Statistic ^a^	HR (95% CI) ^b^	C-Statistic ^b^	Ev+	Ev−	HR (95% CI) ^a^	C-Statistic ^a^	HR (95% CI) ^b^	C-Statistic ^b^
**Death**	Turku	All	90	93	1.79 (1.40, 2.29)	0.707	1.72 (1.32, 2.25)	0.717	85	92	1.02 (0.79, 1.32)	0.486	0.79 (0.60, 1.06)	0.655
	Turku	No residual tumor	21	58	2.12 (1.26, 3.55)	0.648	2.16 (1.20, 3.86)	0.735	21	58	0.86 (0.52, 1.44)	0.532	0.60 (0.35, 1.04)	0.703
	Charité 1	Stage III, no residual tumor	33	17	1.59 (1.08, 2.35)	0.592	1.67 (1.12, 2.47)	0.626	32	16	1.21 (0.82, 1.78)	0.534	1.38 (0.91, 2.08)	0.628
	Charité 2	All	28	76	1.95 (1.31, 2.88)	0.694	1.73 (1.11, 2.71)	0.706	25	74	1.39 (0.94, 2.05)	0.421	1.26 (0.81, 1.98)	0.580
	Charité 3	All	77	66	1.40 (1.12, 1.74)	0.630	1.10 (0.88, 1.38)	0.722	76	64	1.18 (0.92, 1.50)	0.544	1.02 (0.78, 1.32)	0.706
	Charité 3	No residual tumor	42	46	1.40 (1.03, 1.91)	0.621	1.23 (0.91, 1.68)	0.672	42	44	1.21 (0.87, 1.68)	0.550	1.17 (0.84, 1.64)	0.633
**Progression**	Turku	All	122	61	1.40 (1.14, 1.71)	0.644	1.28 (1.04, 1.57)	0.700	118	59	1.35 (1.08, 1.69)	0.585	1.01 (0.76, 1.34)	0.667
	Turku	No residual tumor	34	45	1.28 (0.88, 1.87)	0.524	1.13 (0.71, 1.78)	0.747	34	45	1.29 (0.87, 1.92)	0.418	0.83 (0.50, 1.37)	0.740
	Charité 1	Stage III, no residual tumor	34	16	1.53 (1.02, 2.30)	0.615	1.55 (1.03, 2.32)	0.629	34	14	1.39 (0.95, 2.05)	0.592	1.51 (1.00, 2.28)	0.603
	Charité 2	All	49	55	1.27 (0.90, 1.80)	0.589	1.31 (0.90, 1.90)	0.625	46	53	1.22 (0.88, 1.67)	0.560	1.45 (1.01, 2.09)	0.592
	Charité 3	All	84	59	1.22 (0.99, 1.51)	0.563	1.09 (0.87, 1.37)	0.633	82	58	1.15 (0.91, 1.44)	0.541	1.04 (0.82, 1.32)	0.580
	Charité 3	No residual tumor	58	30	1.32 (1.00, 1.73)	0.567	1.22 (0.92, 1.62)	0.632	57	29	1.09 (0.84, 1.42)	0.476	1.02 (0.78, 1.34)	0.544

Hazard ratios (HRs) are expressed per increase in standard deviation. Ev+, event; Ev−, no event; CI, confidence interval, ^a^ Unadjusted models, ^b^ adjusted with age in all cohorts and additionally by stage in the Turku and Charité 2 studies and additionally by success of tumor removal in the Charité 3 cohort.

## Data Availability

The data presented in this study are available for academic research on request from the corresponding author. The data are not publicly available due to the European General Data Protection Regulation (GDPR).

## Supplementary Materials

The following are available online at https://www.mdpi.com/article/10.3390/cancers13081764/s1, Figure S1: Selection of the prognostic lipid ratio, Figure S2: Risk (%) of progression in 1 year or death in 5 years, Figure S3: Longitudinal analysis of the two-lipid signature and CA-125 in 20 ovarian cancer patients, Table S1: Characteristics of the Turku longitudinal patient cohort, Table S2: Global serum lipidome, CA125 and HE4., Table S3: Prognostic performance of HE4 in the Turku cohort.

## References

[1] Perrotti F., Rosa C., Cicalini I., Sacchetta P., Del Boccio P., Genovesi D., Pieragostino D. (2016). Advances in Lipidomics for Cancer Biomarkers Discovery. Int. J. Mol. Sci..

[2] Zhao G., Cardenas H., Matei D. (2019). Ovarian Cancer—Why Lipids Matter. Cancers.

[3] Hanahan D., Weinberg R.A. (2011). Hallmarks of cancer: The next generation. Cell.

[4] Yan F., Zhao H., Zeng Y. (2018). Lipidomics: A promising cancer biomarker. Clin. Transl. Med..

[5] Ke C., Hou Y., Zhang H., Fan L., Ge T., Guo B., Zhang F., Yang K., Wang J., Lou G. (2014). Large-scale profiling of metabolic dysregulation in ovarian cancer. Int. J. Cancer.

[6] Torre L.A., Trabert B., DeSantis C.E., Miller K.D., Samimi G., Runowicz C.D., Gaudet M.M., Jemal A., Siegel R.L. (2018). Ovarian cancer statistics, 2018. CA Cancer J. Clin..

[7] Karam A.K., Karlan B.Y. (2010). Ovarian cancer: The duplicity of CA125 measurement. Nat. Rev. Clin. Oncol..

[8] Ledermann J.A., Raja F.A., Fotopoulou C., Gonzalez-Martin A., Colombo N., Sessa C. (2013). Newly diagnosed and relapsed epithelial ovarian carcinoma: ESMO Clinical Practice Guidelines for diagnosis, treatment and follow-up †. Ann. Oncol..

[9] Norquist B.M., Brady M.F., Harrell M.I., Walsh T., Lee M.K., Gulsuner S., Bernards S.S., Casadei S., Burger R.A., Tewari K.S. (2018). Mutations in homologous recombination genes and outcomes in ovarian carcinoma patients in GOG 218: An NRG oncology/Gynecologic oncology group study. Clin. Cancer Res..

[10] Monk B.J., Pujade-Lauraine E., Burger R.A. (2013). Integrating bevacizumab into the management of epithelial ovarian cancer: The controversy of front-line versus recurrent disease. Ann. Oncol..

[11] Colombo N., Sessa C., Du Bois A., Ledermann J., McCluggage W.G., McNeish I., Morice P., Pignata S., Ray-Coquard I., Vergote I. (2019). ESMO-ESGO consensus conference recommendations on ovarian cancer: Pathology and molecular biology, early and advanced stages, borderline tumours and recurrent disease. Int. J. Gynecol. Cancer.

[12] Lin H.M., Mahon K.L., Weir J.M., Mundra P.A., Spielman C., Briscoe K., Gurney H., Mallesara G., Marx G., Stockler M.R. (2017). A distinct plasma lipid signature associated with poor prognosis in castration-resistant prostate cancer. Int. J. Cancer.

[13] Cotte A.K., Cottet V., Aires V., Mouillot T., Rizk M., Vinault S., Binquet C., De Barros J.P.P., Hillon P., Delmas D. (2019). Phospholipid profiles and hepatocellular carcinoma risk and prognosis in cirrhotic patients. Oncotarget.

[14] Bachmayr-Heyda A., Aust S., Auer K., Meier S.M., Schmetterer K.G., Dekan S., Gerner C., Pils D. (2017). Integrative Systemic and Local Metabolomics with Impact on Survival in High-Grade Serous Ovarian Cancer. Clin. Cancer Res..

[15] Braicu E.I., Darb-Esfahani S., Schmitt W.D., Koistinen K.M., Heiskanen L., Pöhö P., Budczies J., Kuhberg M., Dietel M., Frezza C. (2017). High-grade serous carcinoma patients exhibit profound alterations in lipid metabolism. Oncotarget.

[16] Shida D., Takabe K., Kapitonov D., Milstien S., Spiegel S. (2008). Targeting SphK1 as a New Strategy against Cancer. Curr. Drug Targets.

[17] Pyragius C.E., Fuller M., Ricciardelli C., Oehler M.K. (2013). Aberrant lipid metabolism: An emerging diagnostic and therapeutic target in ovarian cancer. Int. J. Mol. Sci..

[18] Salminen L., Nadeem N., Jain S., Grènman S., Carpén O., Hietanen S., Oksa S., Lamminmäki U., Pettersson K., Gidwani K. (2020). A longitudinal analysis of CA125 glycoforms in the monitoring and follow up of high grade serous ovarian cancer. Gynecol. Oncol..

[19] Harter P., Sehouli J., Lorusso D., Reuss A., Vergote I., Marth C., Kim J.-W., Raspagliesi F., Lampe B., Aletti G. (2019). A Randomized Trial of Lymphadenectomy in Patients with Advanced Ovarian Neoplasms. N. Engl. J. Med..

[20] Hilvo M., De Santiago I., Gopalacharyulu P., Schmitt W.D., Budczies J., Kuhberg M., Dietel M., Aittokallio T., Markowetz F., Denkert C. (2016). Accumulated metabolites of hydroxybutyric acid serve as diagnostic and prognostic biomarkers of ovarian high-grade serous carcinomas. Cancer Res..

[21] John G., Rustin S., Vergote I., Eisenhauer E., Pujade-lauraine E., Quinn M., Thigpen T., Bois A., Kristensen G. (2011). Definitions for Response and Progression in Ovarian Cancer Clinical Trials Incorporating RECIST 1.1 and CA 125 Agreed by the Gynecological Cancer Intergroup ( GCIG ). Int. J. Gynecol. Cancer.

[22] Folch J., Lees M., Sloane Stanley G.H. (1957). A simple method for the isolation and purification of total lipides from animal tissues. J. Biol. Chem..

[23] Weir J.M., Wong G., Barlow C.K., Greeve M.A., Kowalczyk A., Almasy L., Comuzzie A.G., Mahaney M.C., Jowett J.B.M., Shaw J. (2013). Plasma lipid profiling in a large population-based cohort. J. Lipid Res..

[24] Laaksonen R., Ekroos K., Sysi-Aho M., Hilvo M., Vihervaara T., Kauhanen D., Suoniemi M., Hurme R., März W., Scharnagl H. (2016). Plasma ceramides predict cardiovascular death in patients with stable coronary artery disease and acute coronary syndromes beyond LDL-cholesterol. Eur. Heart J..

[25] Hilvo M., Meikle P.J., Pedersen E.R., Tell G.S., Dhar I., Brenner H., Schöttker B., Lääperi M., Kauhanen D., Koistinen K.M. (2020). Development and validation of a ceramide- and phospholipid-based cardiovascular risk estimation score for coronary artery disease patients. Eur. Heart J..

[26] Takaya H., Nakai H., Takamatsu S., Mandai M., Matsumura N. (2020). Homologous recombination deficiency status-based classification of high-grade serous ovarian carcinoma. Sci. Rep..

[27] Du Bois A., Reuss A., Pujade-Lauraine E., Harter P., Ray-Coquard I., Pfisterer J. (2009). Role of surgical outcome as prognostic factor in advanced epithelial ovarian cancer: A combined exploratory analysis of 3 prospectively randomized phase 3 multicenter trials: By the arbeitsgemeinschaft gynaekologische onkologie studiengruppe ovarialkarzin. Cancer.

[28] Aletti G.D., Dowdy S.C., Gostout B.S., Jones M.B., Stanhope C.R., Wilson T.O., Podratz K.C., Cliby W.A. (2006). Aggressive Surgical Effort and Improved Survival in Advanced-Stage Ovarian Cancer. Obstet. Gynecol..

[29] Messias M.C.F., Mecatti G.C., Priolli D.G., De Oliveira Carvalho P. (2018). Plasmalogen lipids: Functional mechanism and their involvement in gastrointestinal cancer. Lipids Health Dis..

[30] Clifford C., Vitkin N., Nersesian S., Reid-Schachter G., Francis J.A., Koti M. (2018). Multi-omics in high-grade serous ovarian cancer: Biomarkers from genome to the immunome. Am. J. Reprod. Immunol..

[31] Kauhanen D., Sysi-Aho M., Koistinen K.M., Laaksonen R., Sinisalo J., Ekroos K. (2016). Development and validation of a high-throughput LC-MS/MS assay for routine measurement of molecular ceramides. Anal. Bioanal. Chem..

