# Supplementary material for: A Novel Two-Lipid Signature Is a Strong and Independent Prognostic Factor in Ovarian Cancer
Source: Cancers (Basel). 2021 Apr 7;13(8):1764. doi: 10.3390/cancers13081764 (PMC8067677; doi:10.3390/cancers13081764)
Supplement: Supplementary file 1 [file cancers-13-01764-s001.zip › cancers-1139563 supplementary after proof.pdf]

SUPPLEMENTARY DATA

Salminen et al., A novel two-lipid signature is a strong and independent prognostic factor in ovarian cancer

Supplementary Table S1. Characteristics of the Turku longitudinal patient cohort.

Patient	Stage	Treatment Strategy	Residual Disease	Treatment Response	Progression-free survival (months)	Overall Survival (months)
1	IIIC	PDS	0-1cm	CR	27.2	51.3
2	IIIC	NACT	0cm	CR	2.7	31.4
3	IVA	NACT	0-1cm	CR	19.3	49.7
4	IIIC	NACT	>1cm	CR	3	12
5	IIIC	NACT		PD after NACT	0	14.7
6	IVB	NACT	>1cm	PD	0	18.4
7	IIIB	PDS	0cm	CR	19.6	47.7
8	IIIC	PDS	>1cm	PD	0	7.7
9	IIIC	NACT		PD after NACT	0	19.4
10	IVB	PDS	>1cm	CR	20.1	46.6
11	IVB	PDS	0-1cm	PR	3	36.2
12	IIIC	NACT	0-1cm	CR	3	37.1
13	IIIC	NACT	0cm	PD	0	17.6
14	IVB	NACT	0-1cm	PD	0	10.9
15	IIIC	PDS	0-1cm	CR	19.6	72.9
16	IIIC	NACT	0-1cm	CR	4.5	12
17	IIIC	NACT		PD after NACT	0	12
18	IIIC	PDS	0-1cm	CR	27.7	69.2
19	IIIC	NACT	0-1cm	PR	18.1	43.9
20	IIIC	NACT	0-1cm	CR	5.8	28.2

NACT=neoadjuvant chemotherapy, PDS=primary debulking surgery, PD=progressive disease, CR=complete response, PR=partial response

Supplementary Table S3. Prognostic performance of HE4 in the Turku cohort.

	(Sub)group	Ev+	Ev-	HR (95% CI)[a]	C-statistic[a]	HR (95% CI)[b]	C-statistic[b]
**Death**	**All**	41	69	1.12 (0.76, 1.66)	0.561	0.72 (0.46, 1.13)	0.702
	**No residual tumor**	13	42	1.20 (0.59, 2.44)	0.529	0.49 (0.19, 1.26)	0.674
**Progression**	**All**	65	45	1.20 (0.86, 1.68)	0.593	0.94 (0.65, 1.37)	0.698
	**No residual tumor**	22	33	2.07 (1.17, 3.65)	0.659	1.75 (0.86, 3.58)	0.832

Hazard ratios are expressed per increase in standard deviation. Ev+, event; ev-, no event.
[a]Unadjusted models.
[b]Adjusted with age and stage.

573 lipids

↓

Filtering of lipids showing alteration due to ovarian cancer
C-statistic ≥ 0.65 when ovarian cancer patients were compared with benign controls → **167 lipids**

↓

Filtering of lipids showing association with overall and progression-free survival
C-statistic ≥0.6 based on unadjusted Cox regression models → **64 lipids**

↓

Selection of numerator lipid
Lipid with HR > 1 and highest C-statistic in unadjusted Cox regression model
→ **Cer(d18:1/18:0)**

↓

Selection of denominator lipid
Highest C-statistic for the ratio with Cer(d18:1/18:0) from 100 rounds of bootstrapping (C-statistic from 2/3 of the study population in each round)

↓

Lipid ratio	Median C-statistic (95% CI)
**Cer(d18:1/18:0) / PC(O-38:4)**	**0.705 (0.666, 0.731)**
Cer(d18:1/18:0) / PE(O-38:5)	0.699 (0.661, 0.732)
Cer(d18:1/18:0) / PC(O-38:3)	0.696 (0.663, 0.721)
Cer(d18:1/18:0) / PC(O-40:3)	0.694 (0.661, 0.725)
Cer(d18:1/18:0) / LPC(O-22:1)	0.694 (0.657, 0.729)
Cer(d18:1/18:0) / PE(P-34:2)	0.694 (0.658, 0.722)
Cer(d18:1/18:0) / PE(O-36:4)	0.691 (0.659, 0.724)
Cer(d18:1/18:0) / PE(P-36:3)	0.691 (0.653, 0.727)
Cer(d18:1/18:0) / PC(O-36:3)b	0.691 (0.657, 0.721)
Cer(d18:1/18:0) / PI(36:1)	0.690 (0.657, 0.717)

Supplementary Figure S1. Selection of the prognostic lipid ratio. First, only ovarian cancer-associated lipids were left for the analyses, whereafter only those were included in the selection that showed association both with overall and progression-free survival. There was only one lipid showing HR>1, i.e. Cer(d18:1/18:0), which was selected as the numerator lipid. Finally, denominator lipid was selected by calculating C-statistic for all the lipids in ratio with the selected numerator lipid. The highest C-statistic was observed for the Cer(d18:1/18:0) / PC(O-38:4) ratio (in the table 10 lipid ratios with the highest C-statistic are shown).

Stage	Residual tumor	PROGRESSION (1 YEAR)			DEATH (5 YEARS)		
III-IV	> 10 mm	21	30	39	46	75	87
	0 - 10 mm	21	29	38	38	66	79
	0 mm	20	29	37	31	57	71
I-II	All	3	4	6	12	21	33
Lipid ratio quartile		Q1-Q2	Q3	Q4	Q1-Q2	Q3	Q4

Supplementary Figure S2. Risk (%) of progression in 1 year or death in 5 years, based on the lipid ratio quartile, stage and success of tumor removal in surgery in the Turku cohort HGSOC patients.

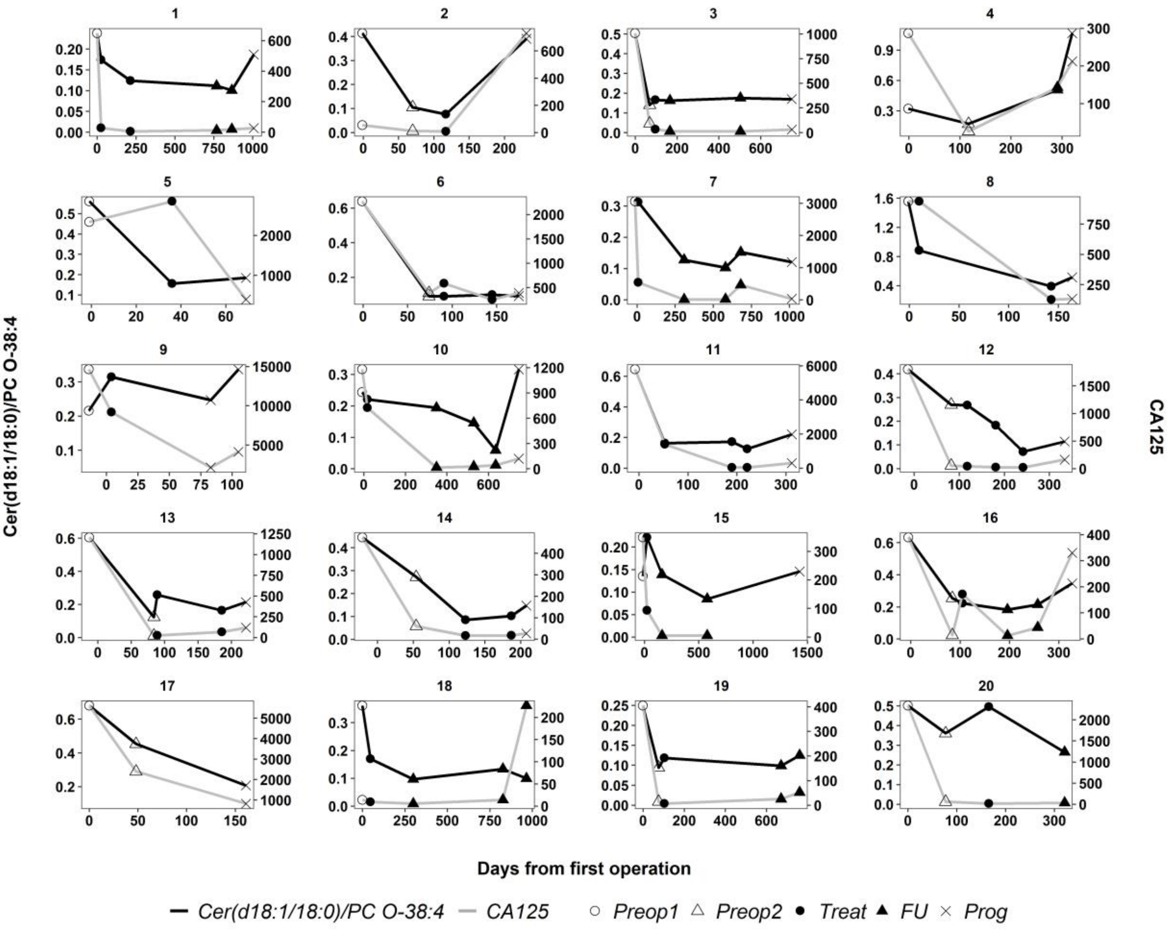

Supplementary Figure S3. Longitudinal analysis of the two-lipid signature and CA-125 in 20 ovarian cancer patients. Patients 18, 19 and 20 did not have samples obtained at progression.
